# Supplementary material for: Azacytidine restores T cell function in AML by modulating DNA methylation
Source: bioRxiv. 2026 Jun 17:2026.06.14.732148. Preprint. [Version 1] doi: 10.64898/2026.06.14.732148 (PMC13308147; doi:10.64898/2026.06.14.732148)
Supplement: Supplement 2 [file NIHPP2026.06.14.732148v1-supplement-2.pdf]

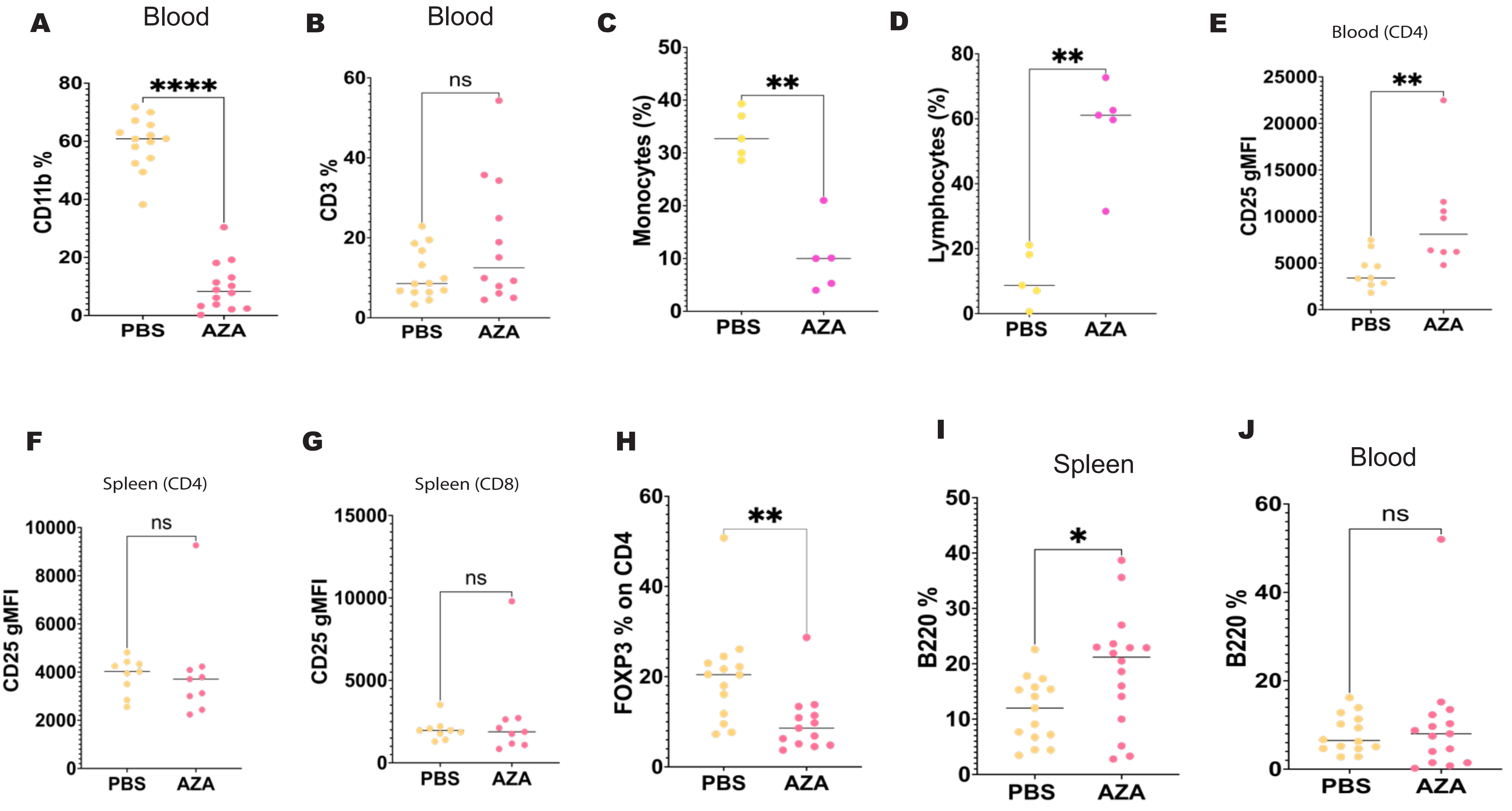
A
Blood
CD11b %
80
60
40
20
0
****
PBS
AZA
B
Blood
CD3 %
60
40
20
0
ns
PBS
AZA
C
Monocytes (%)
50
40
30
20
10
0
**
PBS
AZA
D
Lymphocytes (%)
80
60
40
20
0
**
PBS
AZA
E
Blood (CD4)
CD25 gMFI
25000
20000
15000
10000
5000
0
**
PBS
AZA
F
Spleen (CD4)
CD25 gMFI
10000
8000
6000
4000
2000
0
ns
PBS
AZA
G
Spleen (CD8)
CD25 gMFI
15000
10000
5000
0
ns
PBS
AZA
H
FOXP3 % on CD4
60
40
20
0
**
PBS
AZA
I
Spleen
B220 %
50
40
30
20
10
0
*
PBS
AZA
J
Blood
B220 %
60
40
20
0
ns
PBS
AZA

bioRxiv preprint doi: https://doi.org/10.64898/2026.06.14.732148; this version posted June 17, 2026. The copyright holder for this preprint (which was not certified by peer review) is the author/funder, who has granted bioRxiv a license to display the preprint in perpetuity. It is made available under a CC-BY 4.0 International license.

Supplemental figure legends:

Supplemental figure 1: Aza's impact on tumor burden in AML mouse model.
(A-B) Quantification of the frequency of CD11b+ myeloid cells (A) and CD3+ T cells (B) among live cells in peripheral blood of AML mice treated for three weeks with Aza or PBS, as assessed by flow cytometry. p-value= <0.0001 (A), 0.252 (B).

(C) Frequency of monocytes quantified using automated hematology analyzer at the study end point, comparing Aza and PBS treated mice. p-value= 0.0079.

(D) Frequency of lymphocytes quantified using automated hematology analyzer at the study endpoint, comparing Aza- and PBS-treated mice. p-value= 0.0079.

(E) Geometric mean fluorescence intensity (gMFI) of CD25 expression on CD4+ T cells, measured in the blood after three weeks of PBS or Aza treatment. p-value= 0.0055.

(F) (G) gMFI of CD25 expression on CD4+ T cells (F) (p-value= 0.341) and CD8+ T cells (G) (p-value= 0.863) isolated from spleens of PBS and Aza treated mice after three weeks of PBS or Aza treatment.

(H) Frequency of FOXP3+ cells in CD4+ cells, in blood of Aza- and PBS-treated AML mice. p-value= 0.0033.

(I-J) Frequency of B cells, identified by B220 expression among live cells in the spleen (I) (p-value= 0.0220) and blood (J) (p-value= 0.747), respectively, of AML mice treated for three weeks with Aza or PBS.

Data was analyzed using GraphPad Prism. Statistical significance determined using Mann-Whitney U test. Each data point represents an individual mouse.

A

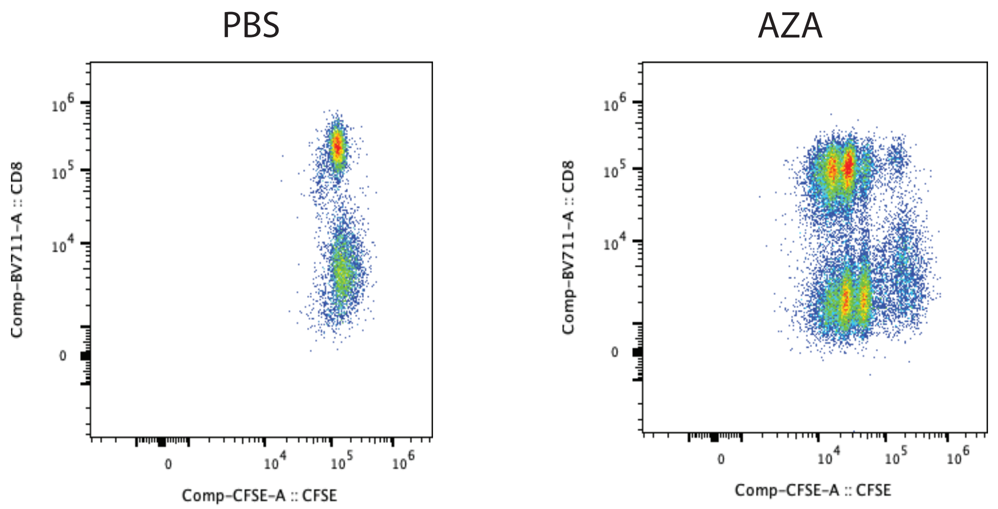

bioRxiv preprint doi: https://doi.org/10.64898/2026.06.14.732148; this version posted June 17, 2026. The copyright holder for this preprint (which was not certified by peer review) is the author/funder, who has granted bioRxiv a license to display the preprint in perpetuity. It is made available under a CC-BY 4.0 International license.

Supplemental figure 2: Proliferation post Aza treatment.

(A) Representative flow cytometry dot plots comparing PBS and Aza treated mice following anti-CD3 stimulation, showing CFSE (x-axis) vs CD8 (y-axis). Plots are gated on live CD3+ T cells.

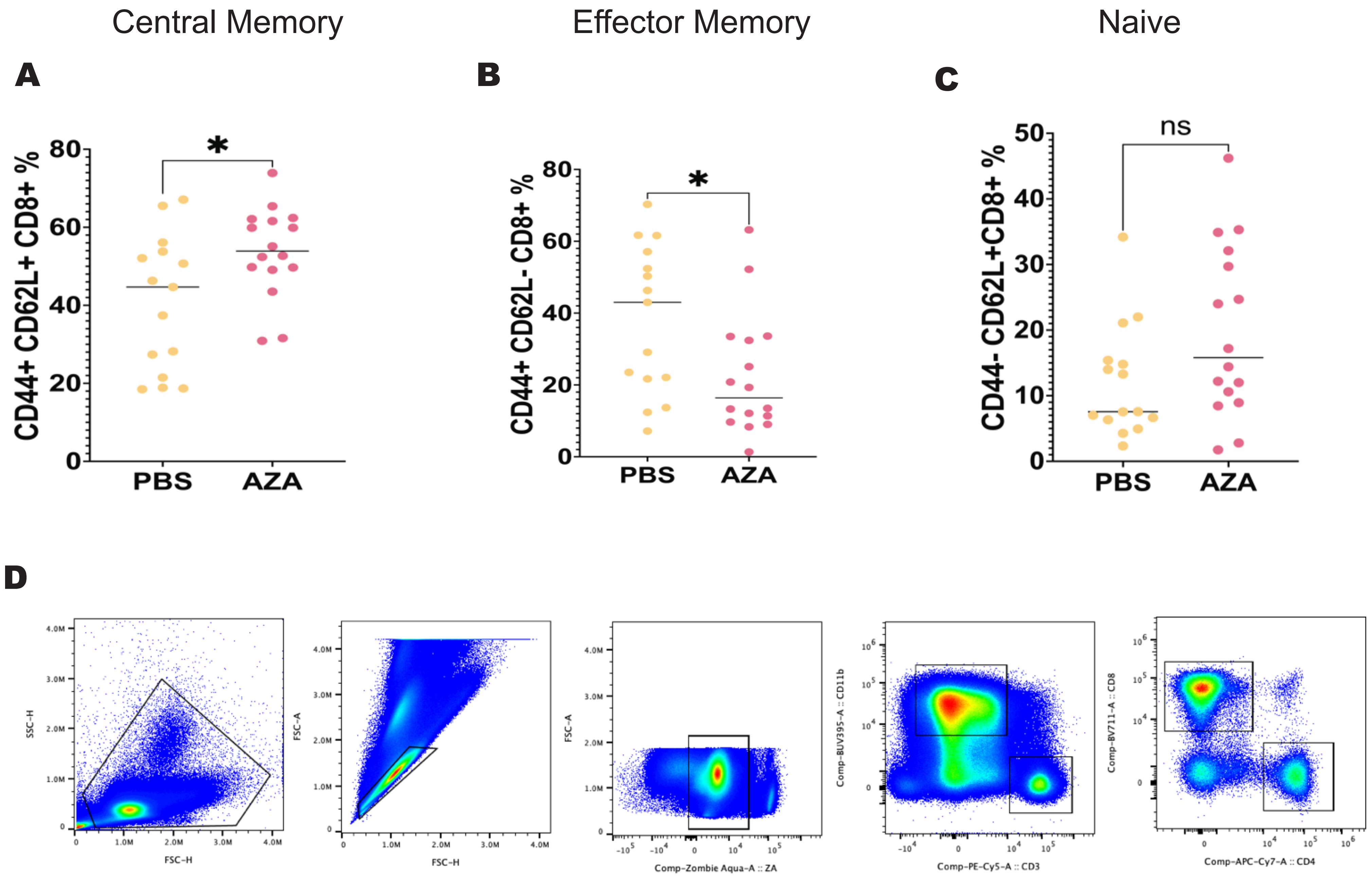
Central Memory
Effector Memory
Naive
A
B
C
D
CD44+ CD62L+ CD8+ %
CD44+ CD62L- CD8+ %
CD44- CD62L+CD8+ %
*
*
ns
PBS
AZA
0
20
40
60
80
10
30
50
SSC-H
FSC-H
FSC-A
Comp-Zombie Aqua-A :: ZA
Comp-BUV395-A :: CD11b
Comp-PE-Cy5-A :: CD3
Comp-BV711-A :: CD8
Comp-APC-Cy7-A :: CD4

bioRxiv preprint doi: https://doi.org/10.64898/2026.06.14.732148; this version posted June 17, 2026. The copyright holder for this preprint (which was not certified by peer review) is the author/funder, who has granted bioRxiv a license to display the preprint in perpetuity. It is made available under a CC-BY 4.0 International license.

Supplemental figure 3: Memory subset changes after Aza treatment in spleens of AML mice.

(A-C) Quantification of central memory (CD44+ CD62L+; A), effector memory (CD44+ CD62L-; B) and naïve (CD44-CD62L+; C) CD8+ T cell subsets in the spleens of AML mice treated with PBS or Aza for three weeks. p-value for central memory is 0.0357, effector memory is 0.033, naïve is 0.0856.

(D) Representative gating strategy used for analyzing flow cytometry data using FlowJo.

Data is pooled from 4 independent experiments. Statistical significance was determined using Mann-Whitney U test. Each data point represents an individual mouse.

A

Comparison (A vs B)	No. Sig DMRs Total	No. Sig Hyper DMRs (more methylation in "A")	No. Sig Hypo DMRs (less methylation in "A")
AZA CD8 vs PBS CD8	3938	1876	2062
AZA CD4 vs PBS CD4	3727	1913	1814

B

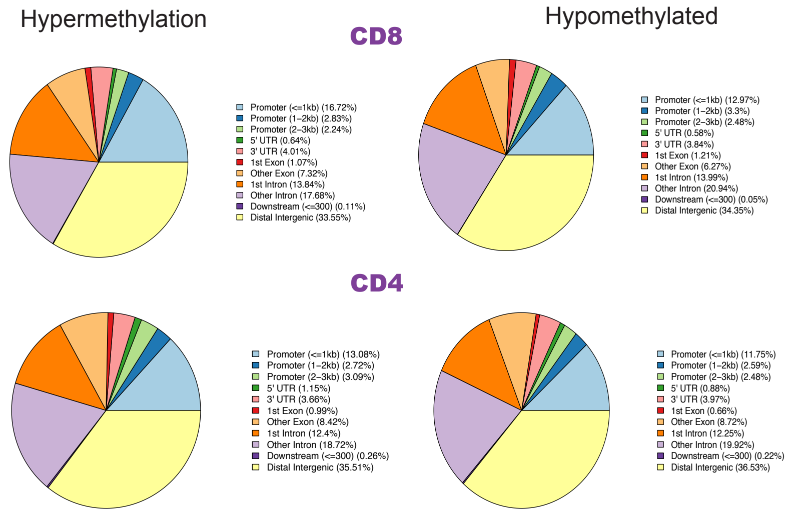

C

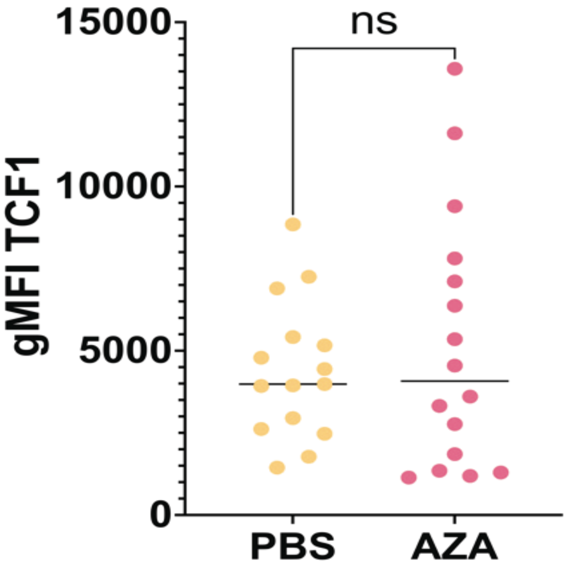

D

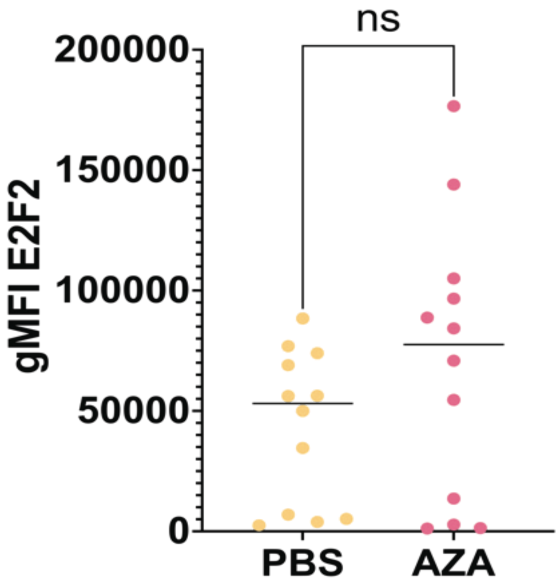

bioRxiv preprint doi: https://doi.org/10.64898/2026.06.14.732148; this version posted June 17, 2026. The copyright holder for this preprint (which was not certified by peer review) is the author/funder, who has granted bioRxiv a license to display the preprint in perpetuity. It is made available under a CC-BY 4.0 International license.

Supplemental figure 4: RRBS revealing hypomethylation changes as well as protein expression changes in spleens post treatment.

(A) Table summarizing the total number of differentially methylated regions (DMRs) identified in CD4+ and CD8+ T cells of Aza or PBS-treated mice.

(B) Pie charts showing the genomic annotation of differentially methylated regions (DMRs) identified in CD8+ (top) and CD4+ (bottom) T cells, categorized by hypermethylated (left) and hypomethylated (right) regions across treatment groups. The DMRs were distributed across multiple genomic regions, including promoters, gene bodies and intergenic regions, consistent with widespread epigenetic remodeling.

(C-D) Geometric mean fluorescence intensity (gMFI) of TCF1 expression (C) (p-value= >0.999) and E2F2 (D) (p-value= 0.2913) measured by flow cytometry in splenic CD8+ T cells from leukemic mice following three weeks of Aza or PBS treatment *in vivo*. Cells were gated on live CD3+ CD8+ T cells prior to analysis. Data is pooled from 3-4 independent experiments.

Statistical significance was determined using Mann-Whitney U test. Each data point represents an individual mouse.
